# Comparative Oncogenomic Analysis of Copy Number Alterations in Human and Zebrafish Tumors Enables Cancer Driver Discovery

**DOI:** 10.1371/journal.pgen.1003734

**Published:** 2013-08-29

**Authors:** GuangJun Zhang, Sebastian Hoersch, Adam Amsterdam, Charles A. Whittaker, Eline Beert, Julian M. Catchen, Sarah Farrington, John H. Postlethwait, Eric Legius, Nancy Hopkins, Jacqueline A. Lees

**Affiliations:** 1 David H. Koch Institute for Integrative Cancer Research, MIT, Cambridge, Massachusetts, United States of America; 2 Bioinformatics Group, Max Delbrück Center for Molecular Medicine, Berlin, Germany; 3 Department of Human Genetics, Catholic University Leuven, Leuven, Belgium; 4 Institute of Neuroscience, University of Oregon, Eugene, Oregon, United States of America; University of Washington, United States of America

## Abstract

The identification of cancer drivers is a major goal of current cancer research. Finding driver genes within large chromosomal events is especially challenging because such alterations encompass many genes. Previously, we demonstrated that zebrafish malignant peripheral nerve sheath tumors (MPNSTs) are highly aneuploid, much like human tumors. In this study, we examined 147 zebrafish MPNSTs by massively parallel sequencing and identified both large and focal copy number alterations (CNAs). Given the low degree of conserved synteny between fish and mammals, we reasoned that comparative analyses of CNAs from fish versus human MPNSTs would enable elimination of a large proportion of passenger mutations, especially on large CNAs. We established a list of orthologous genes between human and zebrafish, which includes approximately two-thirds of human protein-coding genes. For the subset of these genes found in human MPNST CNAs, only one quarter of their orthologues were co-gained or co-lost in zebrafish, dramatically narrowing the list of candidate cancer drivers for both focal and large CNAs. We conclude that zebrafish-human comparative analysis represents a powerful, and broadly applicable, tool to enrich for evolutionarily conserved cancer drivers.

## Author Summary

Cancer is essentially a genetic disease, caused by serial genetic changes including point mutations and chromosome number abnormalities. The latter leads to copy number alterations of many genes. While there are usually thousands of these genetic changes in a given tumor, only a small fraction likely contribute to cancer development. One of the major challenges is to distinguish these cancer “driver” genes from “passenger” mutations that do not contribute to the cancer phenotype. In particular, identifying the driver genes on entire chromosomes that are frequently gained or lost in tumors remains a recalcitrant problem as these alterations contain so many genes. We demonstrate that, because the chromosomal location of genes is highly scrambled between zebrafish and human, the number of passenger genes can be dramatically reduced by comparing the genes in copy number alterations found in zebrafish and human tumors. Thus, our approach dramatically narrows down the list of candidate cancer drivers, and can accelerate discovery of novel cancer drivers and pathways that could inform future targeted therapy and personalized medicine.

## Introduction

The genomes of cancer cells usually contain a large number of aberrations (point mutations, copy number alterations [CNAs], chromosome translocations and epigenetic changes), which include causative genetic alterations (drivers) and a far greater number of genetic events (passengers) that do not influence cancer progression [1]. Identification of cancer drivers will advance our understanding of cancer biology and ultimately enable personalized cancer therapies. However, distinguishing drivers from passengers remains difficult because of the number and variability of genomic alterations in cancer cells.

Copy number alterations are detected by methods including cytogenetics, array comparative genome hybridization (aCGH) and massively parallel sequencing [2]. The sizes of CNAs are variable and range from less than a single gene to entire chromosome changes [3], [4]. Cancer drivers have been successfully identified within recurrent focal CNAs by using functional studies to evaluate all of the candidate genes [5]. In contrast, commonly observed large chromosome or chromosome arm-level CNAs, which are usually caused by aneuploidy, encompass too many genes to allow this approach. Neither improved resolution of genome scanning technology nor increased tumor sample size can fully resolve this problem because many cancer drivers likely occur within large CNAs [6], [7]. Thus there is a critical need in the cancer field to find a way to reduce the number of candidate drivers in these very large CNAs to a number amenable to one-by-one functional testing [4], [8]–[12].

Cross-species comparative oncogenomics is one approach to overcome this obstacle [13], [14]. It is well established that the function of human cancer genes is well conserved in other mammals [15]. Recent large-scale mouse-to-human and dog-to-human comparisons confirmed that evolutionary conservation could be used as a filter to reduce the noise in genomic data sets [16]–[20]. Unfortunately, most mouse tumors exhibit little natural aneuploidy, and have fewer and less variable CNAs than human tumors. This reduces their effectiveness for comparative oncogenomics; although there are some exceptions, including malignant peripheral nerve sheath tumors (MPNSTs) as recently shown by CGH analysis of a small number of tumors [21]. Additionally, conserved syntenic blocks among mammals tend to be very large and thus the efficiency of filtering out passengers is relatively poor. As a result, these inter-mammal comparisons have mostly concentrated on focal CNAs.

We sought to enhance the power of cross-species comparisons by using the more evolutionarily distant zebrafish. Teleost fish and the mammalian lineages separated about 450 million years ago and their respective genomics show a high degree of reshuffling, yielding a much lower degree of conserved synteny between human and zebrafish than between human and mouse [22], [23]. Defining conserved synteny as pairs of genes that are within 100 genes of each other in each species, 90% of syntenic blocks conserved between zebrafish and humans contain 10 genes or fewer, and only 2% contain greater than 30 genes (see [24]and Figure S1). Consequently, the passenger genes that are co-enriched or co-depleted with genuine drivers in CNAs are more likely to differ between human and fish than between human and other mammals.

Importantly, the zebrafish is now well validated as an excellent system in which to model human cancer. Zebrafish offer significant technical advantages due their large number of offspring, tractable genetics and amenability to in vivo imaging and chemical screening [25]. Numerous zebrafish models confirm that the function of core cancer genes, such as *tp53*, *pten*, *nf1*, *nf2*, *Myc*, *Mycn*, mutant *KRAS*, and mutant *BRAF*, is conserved between humans and zebrafish [26]–[33]. Notably, several cancer mutations known to cause particular human tumor types have been shown to can lead to the same tumor types in zebrafish [26], [27], [30], [32], [33]. Moreover, a comparative oncogenomics study of human versus zebrafish T-cell acute lymphoblastic leukemia (T-ALL) successfully identified genes that were shared between focal CNAs in both species [34]. This provides strong justification for zebrafish-human tumor CNA comparisons, at least in the context of tumor types that have low-level aneuploidy. Given this success, we wished to apply this approach to tackle chromosome-arm level CNAs.

We chose to address this question in MPNSTs, a tumor type that in humans displays particularly high levels of aneuploidy and has very poor prognosis. With the exception of a few hereditary susceptibility genes, such as *NF1* and *NF2*, drivers for this cancer type remain largely unknown. This in part reflects the extensive aneuploidy of these tumors and the consequent difficulty in identifying the key changes amongst so much genomic alteration. In zebrafish, MPNSTs are a very rare spontaneous tumor type, but various genetic mutations can predispose fish to develop them including heterozygosity for *nf2a* (albeit at low penetrance), heterozygosity for any one of various *ribosomal protein* (*rp*) genes and homozygosity for an inactivating *tp53* mutation, *tp53^M214K^* [27], [28], [35]. *Rp* heterozygotes and *tp53* homozygotes develop MPNSTs at very high penetrance and tumors from the two genotypes have indistinguishable gene expression patterns. Consistent with this finding, our studies support a mechanistic link between these two MPNST models by showing that tumor cells in *rp* heterozygotes are unable to induce the tp53 protein [36]. Pathologists in multiple laboratories determined that these tumors were MPNSTs based upon both histological analysis and electron microscopy. Similar to human MPNSTs and also MPNSTs in murine genetically engineered models, these tumors consist of spindle cells aligned into stacks and fascicles to form a whirling, storiform pattern [27], [28], [37]–[39]. Moreover, electron microscopy studies indicate that the tumor cells have elongated interdigitating cytoplasmic processes and reduplicated external lamina, morphologic characteristics of nerve sheath differentiation [28]. Additionally, microarray analysis of both *rp* and *p53* tumors indicated high expression of S100 in these tumors [36], which is a common diagnostic marker for MPNSTs.

Importantly, there is some overlap between the initiating genetic lesions seen in the zebrafish MPNSTs and human MPNSTs. As noted above, mutation of one paralog of the human NF2 gene, *nf2a*, can predispose zebrafish to develop MPNSTs, albeit at low penetrance that likely reflects compensation due to the duplication of this gene in zebrafish. Human MPNSTs, including those with mutation of the *NF1* gene, frequently lose the *CDKN2A* gene, encoding both p16 and ARF, which disrupts activation of p53 [40]–[42]. Additionally, recent studies showed that mutation of both zebrafish paralogs of *NF1* accelerates MPNST onset in *p53* mutants [33]. Taken together, these studies suggest that zebrafish MPNSTs share drivers with human MPNSTs.

We previously demonstrated that *rp* and *tp53* mutant MPNSTs both display a high degree of aneuploidy [43]. Specifically, mitotic spreads showed that the chromosome number varied considerably between individual cells within each tumor, with the average trending around 3N [43]. To determine whether zebrafish MPNSTs contain recurrent genomic changes, we conducted a pilot CNA study of 36 tumors and were able to detect both recurrent focal CNAs and preferred whole-chromosome CNAs [43]. Notably, both types of genomic changes are a hallmark of human MPNSTs [6], [44].

Given the limited conservation of synteny between human and zebrafish, we hypothesized that a gene-level comparison of CNAs in zebrafish and human MPNSTs could be employed to reduce the number of candidate cancer drivers on chromosome-arm level CNAs to be analyzed by functional studies. In this study, we stringently defined CNAs in zebrafish MPNSTs through analysis of 147 additional MPNSTs, and compared the preferred changes to ones that are characteristic of human MPNSTs. This comparative approach significantly reduced the number of candidate MPNST driver genes by approximately four-fold.

## Results

### Zebrafish MPNSTs contain preferential chromosome-level and focal copy number alterations

We chose to test the power of zebrafish and human comparative oncogenomics in the context of MPNSTs because the molecular determinants of this tumor type are poorly understood and the extensive aneuploidy makes it a particularly challenging problem. The general strategy of our approach is outlined in Figure S2. Our first step was to construct a high-confidence map of recurrent copy number alterations in zebrafish MPNSTs. Initially, we identified CNAs for individual tumors by comparison of the massively parallel sequencing of DNA taken from fresh tumors versus normal (tail) tissue from the same fish. This latter control was particularly important because it has been shown that portions of the normal zebrafish genome can exhibit fish to fish germline copy number variation [45]. As noted above, the MPNSTs arising within diploid fish have near-triploid genomes [43]. Thus, the copy number calls for the tumor tissue were made relative to this 3N baseline copy number, such that underrepresented chromosomes (“loss”) exist at less than three copies, and overrepresented chromosomes (“gains”) exist at greater than three copies. These zebrafish MPNSTs were isolated from several different genetic backgrounds. 53 came from diploid fish heterozygous for any one of 14 *rp* mutations (on 11 different chromosomes), and 49 were isolated from diploid fish homozygous for *tp53^M214K^*. In addition, given that MPNSTs have a near-triploid copy number [43] and triploid zebrafish are viable [46], we also analyzed 45 tumors from triploid *tp53^M214K^* homozygotes to determine whether starting with a triploid genome would alter the genomic content of the resultant tumors. Interestingly, MPNSTs arising in triploid *tp53^M214K^* homozygotes had a pseudo-triploid chromosome number similar to MPNSTs from diploid fish, arguing strongly that this represents the preferred genomic state of this tumor type. Heat maps of all 147 tumors are shown in Figure S3A and per-sample numerical data is available in Dataset S1 and Dataset S2.

**Figure 1:**
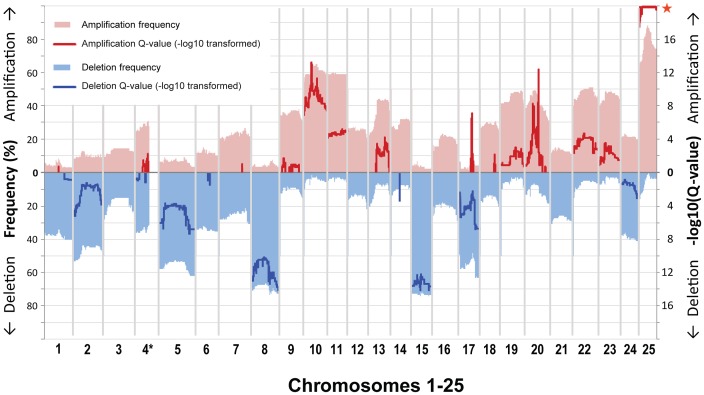
Gene-based frequency and Q-value profiles for gains and losses over the 25 zebrafish chromosomes. Gains and losses are shown in red (top) and blue (bottom). Frequencies (left y-axis, pale red/blue shading) are displayed with respect to a cutoff of 0.2 as used for the GISTIC analysis. GISTIC Q-values (right y-axis, bold red/blue lines) are displayed as −log10-transformed only above a value of 0.6 used as cutoff (corresponding to an untransformed Q-value of 0.25). We did not evaluate gene data for the large portion on chromosome 4 that is characterized by high repeat and GC content (*), and accordingly it is only partially shown.

We next determined which CNAs were recurrent (i.e. found in tumors significantly more frequently than would be expected by chance, given the amount of CNA per tumor). For this, segmented per-sample data for all 147 tumor:normal comparisons were subjected to statistical analysis using the GISTIC algorithm [47] in its JISTIC implementation [48]. Overall, recurrent large-scale CNAs accounted for almost 60% of the zebrafish genome. This analysis confirmed our prior conclusions about the contributions of whole-chromosome alterations [43], and allowed stringent definition of the recurrent alterations. Specifically, all or most of nine different chromosomes (chromosomes 9, 10, 11, 13, 19, 20, 22, 23 and 25) were overrepresented and six chromosomes (chromosomes 2, 5, 8, 15, 17, 24) were underrepresented (Figure 1, Table S1, Dataset S3). With the exception of chromosome 25, large-scale CNAs showed modest amplitudes, which is similar to findings in most human solid tumors [4]. Zebrafish centromeres have only been roughly mapped [49]–[53]. However, a careful examination of the CNAs in each of the individual tumors did not detect any common copy number breakpoints in the chromosomal region that contains each centromere (Figure S3C). This suggests that zebrafish MPNSTs rarely exhibit “arm-level” CNAs, which are a common feature of human cancers [4], [6], [7]. Tumors arising in triploid versus diploid *tp53* mutants did not show any statistically significant difference in the frequency with which any chromosome's copy number was altered (Table S2). This reinforces our conclusion that MPNSTs select for a similar karyotype regardless of the starting ploidy, and validates inclusion of the triploid fish tumors in our overall analysis. Alterations within *tp53* and *rp* MPNSTs also appeared mostly similar, but a statistical analysis (made possible by the large sample size for both genotypes) revealed a slight preference for loss of chromosomes 6, 17, and 24 and gain of chromosomes 11 and 22 in *rp* tumors compared to *tp53* tumors (Table S2). Notably, the *tp53* gene is on chromosome 5; while this chromosome is recurrently underrepresented in zebrafish MPNSTs, this tendency is no more prevalent in *tp53* mutant tumors than *rp* mutant tumors. This is consistent with our prior finding that both mutations exert their tumorigenic effect via a common pathway [36].

**Figure 2:**
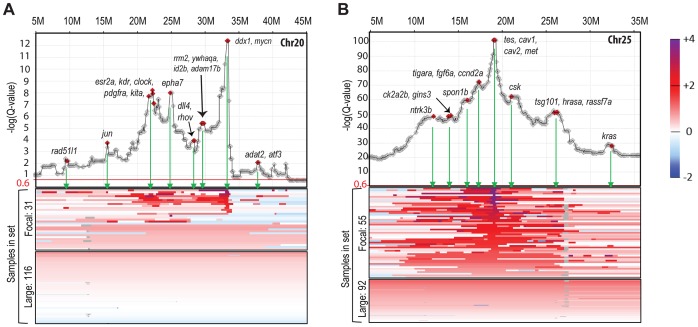
Amplification landscape of focal CNAs reveals sub-peaks. Gene-based Q-value profiles (JISTIC standard mode, −log10-transformed) summarizing the “amplification landscape” on chromosome 20 (A) and chromosome 25 (B), plotted above heatmaps (color scale on the very right) for all underlying 147 zebrafish MPNST samples. For each chromosome, samples are separated into two heatmap panels as indicated to the left of each panel: one for samples with focal and sub-chromosomal alterations (top) and another one for samples with large alterations comprising the entire chromosome (bottom). Within each panel, samples are sorted based on the amplitude at the primary peak (maximum −log10-transformed Q-values), or, for samples with focal alterations not supporting the primary profile peak, by a secondary peak. Diamond markers in profiles show gene locations. Genes with a described role in cancer are highlighted and labeled at the green arrows pointing to the corresponding heatmap regions of focal segment overlaps.

Almost every individual zebrafish tumor displayed a variety of focal CNAs (i.e. affecting less than half a chromosome). Most of the identified focal CNAs spanned less than 10% of the chromosome. Additionally, most were not recurrent. Despite this heterogeneity, we did detect a number of recurrent focal CNAs. These were defined as either JISTIC-determined regions of less than 10 Mb and/or regions that scored in JISTIC's focal mode (see Materials and Methods), which denotes significant recurrence relative to neighboring chromosomal sequences. Importantly, as anticipated, our enlarged sample size detected additional CNAs that were not evident in our previous study [43], and it further refined the boundaries of formerly identified focal changes. In total, we found fourteen recurrent focal gains and three recurrent focal losses (Figure 1, Table S1, Dataset S3). Some of these focal changes overlie large events, and the focal and large alterations point in either the same or opposite directions. For example, focal amplifications are detected at multiple regions of chromosomes 20 and 25, beyond the degree to which the whole chromosome is over-represented, and chromosome 17 contains several small over-represented regions even though it is generally under-represented. In addition, some of the focal CNAs that appear to be a rather large contiguous region (as defined by the algorithm used) have a fine structure that suggests several sub-peaks (local Q-value maxima, Figure 2). Because the Q-values across the entire region score as significant, any part could include driver genes. However, we speculate that the sub-peaks, which in a sense represent minimal overlap regions, may contain higher-probability candidates. Accordingly, we note that these regions often include the zebrafish orthologs of known oncogenes, such as *jun*, *pdgfra*, *kita*, *mycn*, *ccnd2a*, *met*, *hrasa*, and *kras*.

Chromothripsis, a recently described phenomenon of cancer genomes [54], is the catastrophic shattering of chromosomes followed by imperfect fragment rejoining and consequent acquisition of multiple genomic rearrangements. One result of these rearrangements is that a number of segments of a chromosome that were originally non-adjacent become linked and then co-amplified or co-depleted. In CNA analysis (when viewing the sequence of the chromosome in its original order), this presents as an alternation between two or more copy number states along the length of all or part of the chromosome. Evaluation of the copy number data from our 147 tumors identified at least 47 chromosomes that had CNA patterns indicative of chromothripsis (1.3% of all chromosomes). These were observed in both *tp53* and *rp* mutant zebrafish MPNSTs. Two examples are shown in Figure S3E, where the copy number clearly toggles back and forth between two or three different copy number states. While the degree of alteration seems less dramatic than cases reported in human tumors [7], [54], this indicates another similarity in the pathobiology of zebrafish and human cancer. More broadly, our data suggests that chromothripsis may be a hallmark of cancer-associated genomes in all vertebrates.

### Human MPNSTs also contain recurrent CNAs

We next focused our attention on analysis of human MPNSTs. Recently, 23 human MPNSTs in patients with inherited neurofibromatosis type 1 (NF1; heterozygous germline *NF1* mutation) were examined using high resolution aCGH [41] (Figure S3B). Almost half of human MPNSTs develop from neurofibromas in patients with *NF1* mutations and these have been reported to share similar CNA and transcriptome profiles with sporadic MPNSTs [44], [55], [56]. Thus, we believe that this dataset will not be overly biased towards *NF1*-specific cooperating mutations. To enable comparison with our zebrafish data, we re-analyzed this human dataset using the same methods (segmentation, GISTIC). To compensate for the small sample size of human tumors, we analyzed large-scale changes using an increased sensitivity threshold while ensuring that the resulting calls were largely consistent with the previously reported results [41]. In general agreement with prior studies of human MPNSTs [6], [57]–[59], we found that 5 chromosomes or chromosome arms were over-represented and 13 chromosomes or chromosome arms were under-represented (Table 1, Figure S4, Table S3, Dataset S4). Similar to findings in other human solid tumors [4], [6], [7], chromosome (arm)-level changes in human MPNSTs generally exhibited low amplitudes, but appeared at high frequency.

**Table 1:** Overlap of genes in fish and human MPNST gains and losses.

	Chromosome^a^	# of human protein-coding genes in human CNA	Overlap with fish^b^	Number of human genes in comp table (%)	% of human genes in comp table filtered out by fish
Human Gains	7	942	268	617 (65%)	57%
	8	487	128	347 (71%)	63%
	12	271	62	150 (55%)	59%
	15	441	138	303 (69%)	54%
	17	505	54	368 (73%)	85%
	Focals^c^	196	74	144 (73%)	49%
	**Total**	**2840**	**724**	**1929 (68%)**	**62%**
Human Losses	1	1064	332	768 (72%)	57%
	3	254	64	178 (70%)	64%
	4	495	63	326 (66%)	81%
	8	238	74	137 (58%)	46%
	9	214	36	127 (59%)	72%
	10	785	164	551 (70%)	70%
	11	1344	229	789 (59%)	71%
	13	205	22	158 (77%)	86%
	16 region 1	103	3	70 (68%)	96%
	16 region 2	177	7	118 (67%)	94%
	17	475	165	312 (66%)	47%
	18	292	105	196 (67%)	46%
	22	463	108	316 (68%)	66%
	X	376	38	172 (46%)	78%
	Focals^c^	60	4	40 (67%)	90%
	**Total**	**6545**	**1414**	**4258 (65%)**	**67%**

a Human chromosome containing a given recurrent CNA; how much of each noted chromosome is in the recurrent CNA is noted in Table S3.

b The number of human protein-coding genes within the CNA whose fish ortholog is also in a CNA of the same polarity. For a detailed accounting of which fish CNAs contribute to each of these overlaps, see Table S4.

c All focal recurrent gains or losses have been combined here; details by regions are available in Table 2, Table S4, and Dataset S5.

In addition to recurrent large CNAs, we also identified 13 human recurrent focal gains and 7 recurrent focal losses (Figure S4, Table S3, Dataset S4). Similar to the zebrafish tumors, a subset of these human focal changes overlaid large-scale CNAs (chromosomes 7, 9, 17, see Table S3, Dataset S4). Samples displaying CNA patterns indicative of chromothripsis were also present in the human dataset in 44 instances (8.3% of chromosomes amongst all samples). Select examples in which the copy number toggles between two or three states along the length of the chromosome are shown in Figure S3F.

### Zebrafish-human comparative oncogenomics reduces the number of candidate driver genes

To compare our zebrafish and human CNA datasets, we next established a correspondence table of proposed human-zebrafish orthologs represented by Ensembl gene models. These correspondences originated from reciprocal best hits from protein sequence similarity searches (BLASTP), which were further refined using conserved synteny information [24]. This correspondence table covers 20,649 pairwise relationships. Once gene redundancy is eliminated, it comprises 20,216 distinct zebrafish genes and 13,338 distinct human genes. This disparity is due to a number of factors, but chiefly the increased number of paralogs in zebrafish arising from the teleost-specific, whole genome duplication event [60]. As the retention of both paralogs generally indicates some sub-functionalization, either in expression pattern or activity [61], copy number alteration of either paralog could contribute to tumorigenesis in zebrafish. The zebrafish gene count is further inflated because some genes have been erroneously split into two or more adjacent gene models for lack of connecting transcript evidence. Genes unaccounted for in the correspondence table reflect technical difficulties in ortholog assignment, as well as orphan genes [62] in either lineage. These have been excluded from the following oncogenomic comparisons.

#### Comparing all human CNAs to all zebrafish CNAs

**Figure 3:**
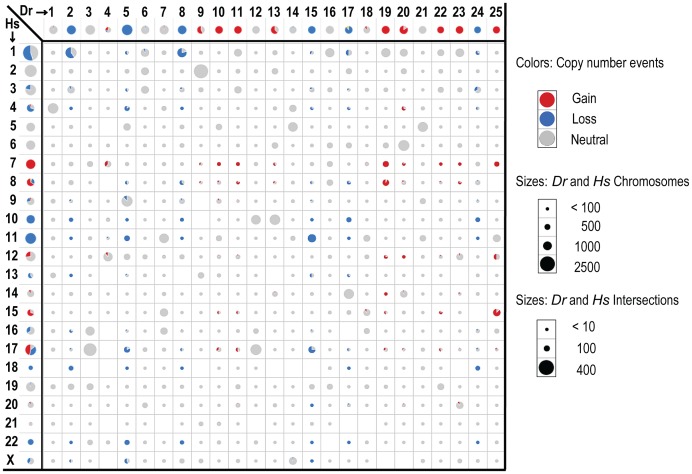
Intersecting human and zebrafish genes by chromosome. Data is shown for genes identified as human-zebrafish orthologs. Axes show genes by chromosome for human (left) and zebrafish (top). The cells in the matrix (intersections) show the overlap of orthologous genes on the individual zebrafish and human chromosomes. Circle and wedge sizes denote the number of genes. Note that the scales differ for the axes versus the intersections, as indicated. The red and blue colors denote recurrent gains and losses for either each individual organism (axes), or the shared changes in both organisms (intersections). The grey shows neutral changes; either no recurrent gain or loss for the per-organism chromosomal gene sets (axes), or lack of concordance (i.e. any combination of gain/loss, gain/neutral, neutral/neutral or loss/neutral) between human and zebrafish orthologs (intersections).

Our first priority was to compare large CNAs between human and zebrafish, with the goal of eliminating likely passengers. We found that only 25% of the genes in human CNA gains were also in zebrafish gains and only 22% of losses were in common between the two species (Figure 3, Table 1, Table S4, Dataset S5). While a non-trivial portion of this reduction reflects the lack of gene representation in the human-zebrafish correspondence table, our imposed restriction that genes must be either gained or lost in both organisms accounted for most of this resolution. For example, of the 487 protein-coding genes (as per Ensembl) on the portion of human chromosome 8 that is gained, 347 are represented in the correspondence table, but only 128 exist within chromosomal gains in zebrafish (Table 1, Table S4, Dataset S5).

Importantly, the human-zebrafish copy number loss intersections included all 3 genes whose hereditary mutation is known to predispose individuals to Schwann cell tumors (neurofibromas, schwannomas and MPNSTs) - *NF1*, *NF2* and *SMARCB1* [63], [64]. Notably, these three genes are all situated in large CNAs, as opposed to focals, and thus would elude analyses centered on focal alterations alone. In addition, our analysis showed that both species lost the *PTEN* tumor suppressor, and *PTEN* inactivation is known to cooperate with *NF1* mutation in MPNST development [65]. Conversely, the copy number gain intersection included quite a number of genes whose overexpression has previously been associated with cancer, such as *BIRC5*, *CCND2*, *CDK6*, *HEY1*, *HGF*, *HSF1*, *KIT*, *MDM2*, *MET*, *NTRK3*, *PDGFR*, *SNAI2*, *TK1*, and *TWIST1*.

#### Comparing human and zebrafish microRNAs

We also screened for miRNAs that were affected by CNAs. For this, we followed a paradigm similar to protein-coding genes, with modifications to accommodate the specifics of miRNA biology. Specifically, rather than using a phylogenetic approach and establishing 1∶1 relationships, we grouped miRNAs based on seed family membership. The resulting human-zebrafish miRNA correspondence list included 89 miRNA seed families for which CNA data was available in both species. We then required that a given miRNA seed family had at least one member in each species that was altered in tumors with the same polarity, and that no other group members were altered in the opposite direction. These stringent criteria identified 8 seed families that were exclusively in gains in both species and 9 exclusively in losses (Table S5). For nearly all of these miRNA seed families, a member miRNA has been implicated in gene expression and copy number alterations across a range of cancer types [66].

#### Genes focally amplified in both human and zebrafish tumors

Many prior cancer studies have concentrated on focal CNAs because of the likelihood that at least one of the encompassed genes will be a cancer driver, as well as the technical feasibility of testing all candidates. We reasoned that any gene detected within both human and fish focal CNAs would be an excellent candidate driver, because such overlaps would be highly unlikely to occur by chance as focally-amplified genes represent only a small percentage of the genome in each species. We found that of 13 recurrent focal gains in human MPNSTs (Table S3), only 4 contained any genes also in zebrafish focal gains (Table 2, Table S4). One of those human focals contained only a single gene (*HIF1A*), and thus our study could not improve the resolution. For the other 3, we found between 28% and 68% of the genes in the human gain were also present in the corresponding fish focal gain (Table 2, Table S4). Thus, even over short regions, the broken synteny between humans and fish can reduce the number of hitchhiker genes. The largest recurrent focal CNA involves a stretch of 34 genes on human chromosome 4; 23 of these genes are also found in a focal gain on zebrafish chromosome 20 (Table 2). In human, this focal event contains a fragile site (FRA4B) and the well-known cancer genes *PDGFR*, *KIT* and *KDR*, which are also in the fish focal gain. Other notable genes found to be focally amplified in both human and fish tumors include *MDM2* and *HIF1A*. In total, 34 genes were found in focal amplifications in both species. In contrast, we did not find any genes to be in focal losses in both species.

**Table 2:** Genes found in focal amplifications in both human and fish MPNSTs, grouped by human focal regions

		Chromosome	
Human Focal Region^a^	Genes	Human	Zebrafish
4q	*DCUN1D4*	4	20
	*LRRC66*	4	20
	*SGCB*	4	20
	*SPATA18*	4	20
	*USP46*	4	20
	*RASL11B*	4	20
	*SCFD2*	4	20
	*FIP1L1*	4	20
	*LNX1*	4	20
	*GSX2*	4	20
	*PDGFRA*	4	20
	*KIT*	4	20
	*KDR*	4	20
	*SRD5A3*	4	20
	*TMEM165*	4	20
	*CLOCK*	4	20
	*NMU*	4	20
	*EXOC1*	4	20
	*CEP135*	4	20
	*KIAA1211*	4	20
	*AASDH*	4	20
	*PPAT*	4	20
	*PAICS*	4	20
12q region #1	*CNTN1*	12	4
	*PDZRN4*	12	4
	*GXYLT1*	12	4
	*YAF2*	12	4
	*ZCRB1*	12	4
12q region #2	*MDM1*	12	4
	*NUP107*	12	4
	*SLC35E3*	12	4
	*MDM2*	12	4
	*CPM*	12	4
14q region #2	*HIF1A*	14	20

a Human focal regions are delineated in Table S3.

#### Genes in focal CNAs in one species but in large CNAs in the other

It is notable that most of the recurrent focal CNAs within either human or zebrafish tumors did not include any genes that were also in focal CNAs in the other organism. Focal CNAs are likely to be influenced by fragile sites and other unstable regions, only a subset of which are evolutionarily conserved. Therefore, it seemed that the focal-to-focal comparison could overlook genuine cancer drivers. Thus, we extended our analysis to identify genes that were affected in focal CNAs in one species and in large CNAs in the other. We initiated this analysis using human tumors as the source of the focal CNAs. Notably, eleven of the thirteen human focal gains had some overlap with the total set of zebrafish gains. Of the 204 genes on these human focal gains, only 89 are present in zebrafish MPNST recurrent gains (55 exclusively in large fish CNAs and 34 that were in fish focals as noted above). Additionally, only two of the seven human focal losses contained genes also lost in zebrafish MPNSTs, and the overlap contains only 4 of the 55 genes on these human CNAs (Table S4, Dataset S5). For both gains and losses, these commonly altered genes might be prioritized over other genes in the human focal CNAs for functional testing.

Given the success of this expanded analysis, we also tested whether we could enrich for likely candidate drivers in large human CNAs by intersecting them with focal CNAs of the same directionality in zebrafish. Of the 2646 human genes present in large copy number gains, 1785 had identifiable zebrafish homologs but only 159 of these were found in recurrent focal amplifications in the zebrafish tumors. Because we found fewer focal losses than gains in the zebrafish tumors, only 14 of the genes within large human losses mapped to focal regions of loss in zebrafish (Table S4, Dataset S5). Both cases may provide biologically important candidates to account for the large gains or losses in human tumors.

#### Chromosome arm-level candidates can be functionally validated

A systematic identification of cancer drivers requires essentially two phases: an effective screening phase to generate candidate genes, and a functional validation phase. Our comparative oncogenomics approach represents a strategy for the first phase. As an example of the functional validation of candidates, we conducted genetic tests on three genes for which we had insertional mutants in house [67]. These include a candidate driver and two putative passenger genes.

**Figure 4:**
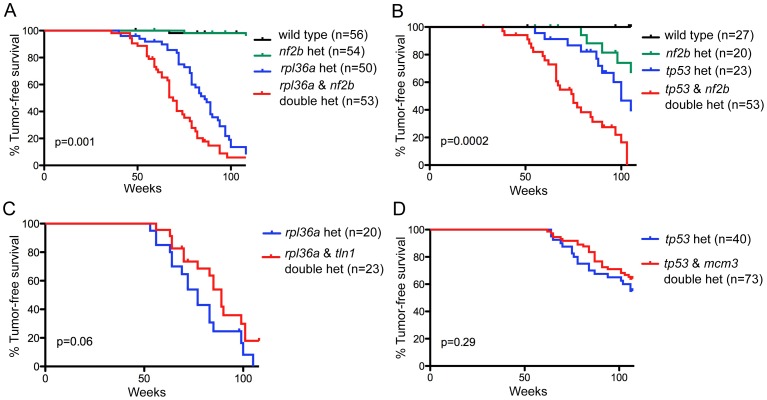
Functional testing of candidate driver gene *nf2* and putative passenger genes. (A,B) *nf2b* loss cooperates with *rp* or *tp53* mutation to promote MPNST development. Kaplan-Meier curves showing tumor-free survival of cohorts of single and double heterozygotes derived from (A) *rpL36a^+/−^*×*nf2b* ^+/−^ crosses and (B) *tp53^M214K/+^*×*nf2b^+/−^* crosses. (C, D) Neither *tln1* nor *mcm3* mutations affect tumor onset in zebrafish MPNST models, suggesting that these genes are passengers in CNAs. Kaplan-Meier curves showing tumor-free survival of cohorts of single and double heterozygotes derived from (C) *rpL36a^+/−^*×*tln1^+/−^* and (D) *tp53^M214K/M214K^*×*mcm3* ^+/−^. Fish from all crosses were genotyped by PCR for each relevant mutation at 6–8 weeks of age and housed segregated by genotype. In all panels, the numbers of fish of each genotype are shown in parenthesis, and the p values between the *rp* or *tp53* single heterozygotes and the double mutants are shown.

The candidate driver was *NF2*, a gene whose loss-function mutation is known to cause human schwannomas. Importantly, our analysis detected *NF2* within large CNAs in both human and zebrafish tumors (Figure S5). In human, *NF2* is on chromosome 22, which is under-represented as a whole. Zebrafish have two paralogs of *NF2*, *nf2a*, which is on an underrepresented zebrafish chromosome (5), and *nf2b*, which is on a neutral chromosome (21). If the *nf2a* and *nf2b* paralogs have similar roles, we reasoned that loss of either might cooperate with the initiating *rp* or *tp53* mutations to promote MPNST development. No *nf2a* mutant currently exists, but we previously identified an *nf2b* zebrafish mutant that has a weak tumor phenotype [27]. To test if this could synergize with the *rp* or *tp53^M214K^* mutations, we intercrossed these lines. In both cases, double heterozygotes developed MPNSTs faster than any of the sibling single heterozygotes, affirming *nf2* as a valid MPNST driver (Figure 4A, B). These findings fit with prior reports that loss of murine *Nf2* and *Trp53* cooperate to yield MPNST [68]. In parallel, we also tested two genes that were identified as putative passengers, based on the fact that they were gained in fish tumors but either lost (*tln1*; zebrafish chromosome 10, human chromosome 9) or not recurrently altered (*mcm3*; zebrafish chromosome 20, human chromosome 6) in human MPNSTs (Figure 4C, D). Consistent with our designation as likely passengers, the heterozygous mutation of these genes had no significant impact on the development of MPNSTs resulting from *rp* or *tp53^M214K^* mutation. We believe that this general methodology can employed to systematically screen the identified candidate drivers.

## Discussion

Our prior study of 36 zebrafish MPNSTs established the presence of aneuploidy and the preferential gain or loss of certain chromosomes [43]. Here, through the analysis of a much larger sample size, we can now assign statistical significance for these changes and conclude that 9 chromosomes are preferentially gained and 6 chromosomes are preferentially lost in these tumors (Table S1). In most cases, these preferences were found in MPNSTs that had been initiated by either *rp* or *tp53* mutations. However, statistical analysis suggests that slight differences may exist for a subset of chromosomes, (Table S2). We note that most of the large-scale CNAs in our zebrafish tumors include entire chromosomes. However, we do find exceptions to this rule, and these CNAs typically affect the central portions of chromosomes, as opposed to the ends. This is somewhat surprising, given that zebrafish chromosomes are predominantly metacentric or submetacentric [43], [69], much like human chromosomes. We speculate that this reflects differences in chromosome breakability between zebrafish and human.

The substantial number of zebrafish MPNST samples also allowed for an accurate assessment of focal CNAs. In addition, we established fine structure for some of the CNA regions, especially for the amplified regions, through changes in GISTIC scores (G-scores) and significance values (Q-values) occurring beyond the simple statistical significance cutoff. These focal significance peaks represent minimal overlapping regions within the context of already statistically significant CNAs, and likely encompass higher-probability candidates. Consistent with this notion, we note that most of these focal peaks contained known oncogenes such as *hrasa*, *kdr*, *kita*, *kras*, *met*, *mycn*, and *pdgfra*.

Comparative oncogenomics is already well validated as a successful strategy to identify cancer drivers [13], [14]. To date, these studies have been primarily limited to analysis of focal CNAs. However, it is clear that many of the large-scale copy number aberrations in solid tumors affect entire chromosomes, chromosome arms, or large portions thereof. Such changes are shared by many types of solid tumors [4], [70]. More importantly, they have been associated with poor prognosis in multiple human tumor types [71]–[77], including in the case of MPNST [44], arguing that they must contain cancer drivers. These large chromosomal CNAs have been hypothesized to reflect the selective advantage of simultaneously targeting multiple cancer drivers [78]. Despite widespread appreciation that whole chromosome and chromosome-arm-sized CNAs must contain important cancer drivers [4], [8]–[12], identification of drivers in these large CNAs has remained a challenge as they simply contain too many genes for one-by-one functional characterization. A reduction of the number of candidate genes to be functionally analyzed would surely make such gene identification more practical, and this is the goal we pursued.

We postulate that zebrafish-human comparative oncogenomics provides a unique opportunity to address chromosome arm-level CNAs because human and fish genomes are effectively “scrambled” relative to each other due to the long evolutionary separation between human and zebrafish [22]. To show this, we established a reliable human-fish gene comparison list that contains 13261, or approximately two-thirds, of human protein-coding genes. This ortholog-based approach may exclude some human cancer genes (as one example, we note that the locus encoding p14ARF is absent in zebrafish), but it places the focus on evolutionarily related genes that are likely to conserve biological function. Using this list, we nominated human genes as candidate drivers if their copy number changed in the same direction as one or more of the zebrafish paralogs. This allowed us to reduce the number of candidate driver genes in the human MPNST CNAs by roughly four-fold. This reduction is comparable to that expected by chance, based on the relative fractions of the human versus zebrafish genomes that are recurrently gained or lost in MPNSTs. As the number of passenger genes is generally thought to greatly exceed the number of genuine cancer drivers, this level of enrichment, and not greater, is the anticipated result. We believe that this underscores the challenge - essentially searching for a needle in a haystack – and highlights how the poor synteny between human and zebrafish has such a strong winnowing effect. While we believe that our list of co-gained and co-lost genes still contains far more passengers than drivers, we note that removing 75% percent of the passenger genes in large CNAs is a significant step towards homing in on the true drivers, making it feasible to functionally test the remaining candidates.

As proof that the retained genes include genuine drivers, we note that the list of genes recurrently lost in both human and fish MPNSTs includes four tumor suppressors, *NF1*, *NF2*, *SMARCB1* and *PTEN*, that are strongly associated with the development of human Schwann cell tumors [63]–[65]. Similarly, the list of co-gained genes includes many genes (e.g. *CCND2*, *ETV6*, *HGF*, *HSF1*, *KIT*, *MDM2*, *MET* and *PDGFR*) whose overexpression and/or gain-of-function mutation are associated with a various human solid tumors, including MPNST. In particular, *MET* has been recently identified as a driver and potential therapeutic target in human MPNSTs [79], *Hsf1* has been shown to be overexpressed and required for ras pathway activation and MPNST development following *Nf1* loss in mice [80], and inhibition of KIT and PDGFR impedes the proliferation of schwannoma and MPNST cell lines and the development of xenograft-derived plexiform neurofibromas [81]–[83].

The reductive power of our analysis is illustrated by consideration of human chromosome 17q, which is amplified frequently in human MPNST, and somewhat in other tumor types. The recurrently affected region includes over 500 genes, precluding systematic gene-by-gene testing. Previous studies had flagged some preferred candidates (e.g. *TOP2A*, *ETV4*, *BIRC5*, *JMJD6*, *SEPT9*, and *SOCS3*) on the basis of mRNA levels in MPNST samples and known biological function [58], [84]. Our comparative analysis identified only 54 of the human 17q genes as being recurrently gained in zebrafish MPNSTs. We believe that this is a tractable number for systematic evaluation for cancer driver function (see below). Notably, of the previously highlighted candidates, only *birc5b* is also gained in zebrafish tumors. Subsequent to the completion of our analysis, it was reported that knockdown, or chemical inhibition, of BIRC5 suppresses growth of MPNST cell lines *in vitro* and xenografts *in vivo* [85].

We also looked carefully at the recurrent focal CNAs identified in the zebrafish MPNSTs, because focal-focal comparisons have been highly effective when comparing tumors from humans with those of other mammals, such as mouse and dog [16]–[20]. In stark contrast to these inter-mammalian comparisons, we found that there was very little concordance between human and zebrafish focals; essentially no overlaps were observed for losses and only a few overlapping genes were identified for gains. Notably, the co-gained regions included a small array of genes (human chromosome 4, zebrafish chromosome 20) that contains *KDR*, *PDGFR* and *KIT*; three genes identified as cancer drivers and potential drug targets in human MPNSTs [81]–[83]. We hypothesize that the dearth of shared focal alterations between human and zebrafish reflects differences in chromosome breakability in these two organisms. Breakability is a function of unstable regions, such as fragile sites and segmental duplications, and recent studies show that human focal CNAs are enriched around such unstable regions [86]. Accordingly, the *KDR/PDGFR/KIT* region on human chromosome 4 is known as a rare fragile site (FRA4B). Thus, we predict that the presence or absence of cross-species focal-focal conservation will be largely determined by the evolutionary conservation fragile sites. Importantly, the lack of cross-species conservation does not rule out the possibility that the species-specific recurrent focal CNAs may carry cancer drivers. To capture these candidates, we looked for the overlap of focal CNAs in one species with large CNAs in the other. This analysis yielded few intersections for losses, but identified about 200 genes for gains that likely represent higher-probability driver candidates.

We were also able to apply human-zebrafish comparisons to the identification of cancer relevant miRNAs. Using stringent search criteria (see results) we identified a handful of miRNAs as very strong candidate drivers (some when lost, some when gained). Notably, nearly all of the identified miRNA seed families have been previously associated with cancer, in some cases causally, e.g. loss of miR-15 and miR-16 [87]. Moreover, one of the microRNA families that we found to be amplified in both species, miR-10, has specifically shown to be overexpressed in NF1-associated MPNSTs, and its inhibition slowed cell proliferation in cell lines derived from such tumors [88].

CNA analysis alone cannot pinpoint individual driver genes, especially when entire chromosomes are recurrently gained or lost. Our comparative oncogenomics approach shrinks the candidate lists dramatically, identifying about 700 commonly gained and 1400 commonly lost genes. Additionally, a focus on higher-probability candidates - those that are in focal alterations in at least one of the two species – further reduced this list to about 250 commonly gained genes. We believe that this is a sufficient small number to allow systematic testing, for example by siRNA screening in human cell lines for transformation-associated phenotypes *in vitro* and tumorigenic ability in xenotransplants. Additionally, our *in vivo* studies show that zebrafish can be used to both validate genuine cancer drivers, as exemplified by our analysis of *nf2b*, and rule out passenger mutations. We believe that the zebrafish has unique features that would greatly enable the testing of large candidate numbers including relatively cheap cost, large clutch size and, most important, the well advanced zebrafish community effort to recover mutants for every gene [89].

In conclusion, our study makes the case that a comparative oncogenomics approach has the potential to overcome a longstanding barrier in cancer research, the aneuploid karyotype, that has by and large remained recalcitrant to systematic analysis owing to the large number of genes simultaneously affected. This provides a new way to mine human cancer CNA data from a comparative perspective, which could accelerate the rate of cancer driver discovery by reducing the number of genes to be tested in functional studies. In principle, the methodology employed here can be readily applied to other cancer types or be expanded to incorporate additional vertebrate species, thus establishing a phylo-oncogenomic basis for analysis.

## Materials and Methods

### Ethics statement

The protocol for the collection and analysis of human tumor samples was approved by the local ethical committee of the University Hospitals Leuven. All animals were housed in AAALAC-approved facilities and maintained according to protocols approved by the Massachusetts Institute of Technology Committee on Animal Care.

### Zebrafish lines and tumor onset analysis

The tumor-prone zebrafish lines carrying either the *tp53^M214K^* point mutation or insertional mutations in multiple ribosomal protein genes (*rpL13^hi1016^*, *rpL14^hi823^*, *rpL35^hi258^*, *rpL36^hi1807^*, *rpL36a^hi10^*, *rpL7^hi1061^*, *rpS3a^hi1290^*, *rpS5hi^577b^*, *rpS5^hi1364a^*, *rpS7^hi1034b^*, *rpS8^hi1974^*, *rpS11^hi2799^*, *rpS15a^hi2649^*, *rpS18^hi1026^*, and *rpS29^hi2903^*) have been described previously [27], [28]. Stocks were maintained as described previously and genotypes were determined by PCR at 8 to 18 weeks of age as described in [67]. Of the zebrafish homozygous for the *tp53^M214K^* point mutation, half were triploid and were made according to previously published methods [46]. Ploidy was determined by measuring DNA content of fish tail cells using propidium iodide (40 µg/ml) staining-based FACS analysis. Fish heterozygous for insertional alleles of *nf2a^hi3332^*, *mcm3^hi3068^* and *tln1^hi3093^* [67] were mated to fish heterozygous for *rpl36a^hi10^* or heterozygous or homozygous for *tp53^M214K^* to obtain sibling single and double heterozygotes for tumor onset experiments. Wild type fish, single heterozygotes, and double heterozygotes arising from these crosses were identified by PCR genotyping [28], [67] at 6–8 weeks of age, and siblings of different genotypes were housed in adjacent tanks at similar densities to minimize environmental differences. Fish were euthanized at first observation of protruding tumors or other signs of ill health, and the presence of MPNSTs in euthanized fish was confirmed by histology.

### Genomic DNA isolation, Illumina sequencing and data processing (zebrafish MPNST samples)

For every tumor, DNA was isolated from macroscopically dissected tumors and separately from normal (tail) tissue from the same fish. Based upon this paired design, CNA calls for all tumors could be determined relative to the genome of the individual fish in which it arose (Dataset S1 and Dataset S1b). Genomic DNA isolation was performed as described previously [43]. Generally, sequencing and data processing was similar as described in [43], with some differences in detail (see Text S1). The zebrafish sequencing data reported in this paper have been deposited in the NIH GEO database (accession no. GSE38397).

### Array-CGH (aCGH) data processing (human MPNST samples)

Normalized aCGH data (Agilent Feature Extraction output) for 23 human MPNST samples generated previously [41], ArrayExpress database Experiment (ID: E-MEXP-3052) was converted from log10 to log2 and submitted to the circular binary segmentation algorithm [90] as implemented in the BioConductor package DNAcopy (v1.16.0), and processed with the following key parameter settings: with smoothing, undo.SD = 1.

### GISTIC (JISTIC) analysis

To determine recurrent CNAs, segmented data from both zebrafish (sequencing) and human (aCGH) MPNSTs was subjected to statistical analysis using the GISTIC algorithm [47] as implemented in the JISTIC software [48]. JISTIC runs were performed in both standard and “focal” mode. Evaluation of JISTIC results (G-scores, Q-values) comprised an additional layer of manual curation, resulting in a final set of binary calls (yes or no) for recurrent large and focal copy number gains and losses (Dataset S3 and Dataset S4, Table S1 and Table S3). Specific details regarding the JISTIC runs and the manual calls are documented in Text S1.

### Human-zebrafish protein coding gene orthologous table construction and comparison

High-confidence human-zebrafish gene correspondences were established based on the approach described in [24], taking advantage of conserved synteny as a guiding principle for identifying evolutionary ortholog pairs, where possible. Only genes with Ensembl protein identifiers (release 61) mapping to assembled zebrafish chromosomes 1–25 and to human chromosomes 1–22 and X were considered. The details of the approach are described in Text S1.

### Human-zebrafish miRNA homologous table construction and comparison

Only genes of Ensembl gene biotype “miRNA” (release 61) from assembled zebrafish chromosomes 1–25 and from human chromosomes 1–22 and X were considered. Human and zebrafish miRNA genes also present in miRBase [91] (662 for human, 315 for zebrafish) were then matched using their miRBase identifiers. Matching was performed based only on the central, numeric part of the identifiers (which denotes a particular miRNA family), resulting in 89 correspondence groups containing one or more miRNAs from both human and zebrafish (Table S5B). These groups were then searched for cases where at least one member miRNA from each species was in a recurrent CNA of a certain polarity, with no member miRNAs in either species being in a recurrent CNA of the opposite polarity.

## Supporting Information

Dataset S1 Gene-based copy-number status (per-sample segment values) in 94 zebrafish MPNST samples from *tp53* mutant fish.
(XLSX)

Dataset S2 Gene-based copy-number status (per-sample segment values) in 53 zebrafish MPNST samples from *rp* mutant fish.
(XLSX)

Dataset S3 Gene-based JISTIC results and final calls regarding recurrent gains and losses based upon the 147 tumors whose data is contained in Datasets S1 and S2. The row identity is identical in Datasets 1–3 so that columns from Datasets S1 and S2 that contain individual tumor data can be copy/pasted into this file if the reader wishes to do so.
(XLSX)

Dataset S4 Gene-based copy-number status in 23 human MPNST samples, comprising per-sample segment values, JISTIC results, and final calls.
(XLSX)

Dataset S5 Protein-coding genes gained or lost in both human and zebrafish MPNSTs.
(XLSX)

Figure S1 Distribution of sizes of syntenic blocks conserved between zebrafish and human. The histogram shows the number of conserved syntenic blocks containing 2–10, 11–20, 21–30 etc. genes. For this purpose, two genes are considered syntenic if they are within 100 genes from each other in both species.
(PDF)

Figure S2 Outline of experimental approach as described in the text.
(PDF)

Figure S3 Overview and highlights of zebrafish and human MPNST CNA data. (A) Heatmap showing an overview of the CNAs for 147 zebrafish MPNST samples over chromosomes 1–25. The panel is subdivided into samples based on *tp53* mutations (94 samples, top) and *rp* mutations (53 samples, bottom) (B) Heatmap showing an overview of the CNAs for 23 human MPNST samples over chromosomes 1–22 and X. (C) As illustrated by three examples (heatmaps for complete chromosomes 7, 9, and 24), zebrafish CNA data from MPNST samples do not suggest the existence of recurrence patterns consistent with chromosomal arms. Dashed line boxes indicate the windows within which the centromeres for these three chromosomes have been genetically mapped. (D) By contrast, and in agreement with previous studies in multiple cancer types, CNA data from human MPNSTs often reveal variability linked to chromosomal arms (chromosomes 1 and 7 shown as examples) (E, F) Repeated alternations between two or more copy number states have been described as a hallmark of chromothripsis. Such alternations, toggling either between copy number loss and neutral, between different levels of amplification, or between copy number loss and copy number gain, can be seen in the heatmaps of sample chromosomes from individual zebrafish (E) or human (F) tumors. Chromothripsis can include either a portion of a chromosome or the entire chromosome, as indicated by the brackets above each example.
(PDF)

Figure S4 Gene-based frequency and Q-value profiles for gains (top, red) and losses (bottom, blue) over human chromosomes 1–22 and X, based on 23 MPNST samples. Gains and losses are shown in red (top) and blue (bottom). Frequencies (left y-axis, pale red/blue shading) are displayed with respect to a cutoff of 0.2 as used for the GISTIC analysis. GISTIC Q-values (right y-axis, bold red/blue lines) are displayed as −log10-transformed only above a value of 0.2 used as human-specific cutoff. The −log10-transformed Q-value for the deletion on chromosome 9 marked by a star is clipped to fit the figure and actually peaks at 20.6.
(PDF)

Figure S5 Human and zebrafish *NF2* genes reside in large, not focal, CNAs. Heat maps of human (A) and zebrafish (B) CNA data showing 10 MB windows centered on *NF2* loci. In each panel, samples are sorted top-to-bottom by decreasing deletion amplitude at the respective *NF2* locus indicated in the center (green line). Blue and red bars at the right side of each panel indicate which samples, with respect to the *NF2* locus, were actually counted as losses (blue) or gains (red) in our JISTIC analysis.
(PDF)

Table S1 Listing of GISTIC-defined recurrent large and focal gains and losses in zebrafish MPNSTs.
(XLSX)

Table S2 P-values from t-Tests (two-tailed, homoscedastic) comparing per-chromosome median values of different subgroups of zebrafish MPNST samples.
(XLSX)

Table S3 Listing of GISTIC-defined recurrent large and focal gains and losses in human MPNSTs.
(XLSX)

Table S4 Number of genes gained or lost in both fish and human MPNST by chromosomal region.
(XLSX)

Table S5 Gains and losses of microRNA families in human and zebrafish MPNSTs.
(XLSX)

Text S1 Supporting Materials and Methods.
(DOCX)
