# Supplementary figures and images for: Comparative Oncogenomic Analysis of Copy Number Alterations in Human and Zebrafish Tumors Enables Cancer Driver Discovery
Source: PLoS Genet. 2013 Aug 29;9(8):e1003734. doi: 10.1371/journal.pgen.1003734 (PMC3757083; doi:10.1371/journal.pgen.1003734)

Figure S1

**Dre vs. Hsa (Window Size = 100)**

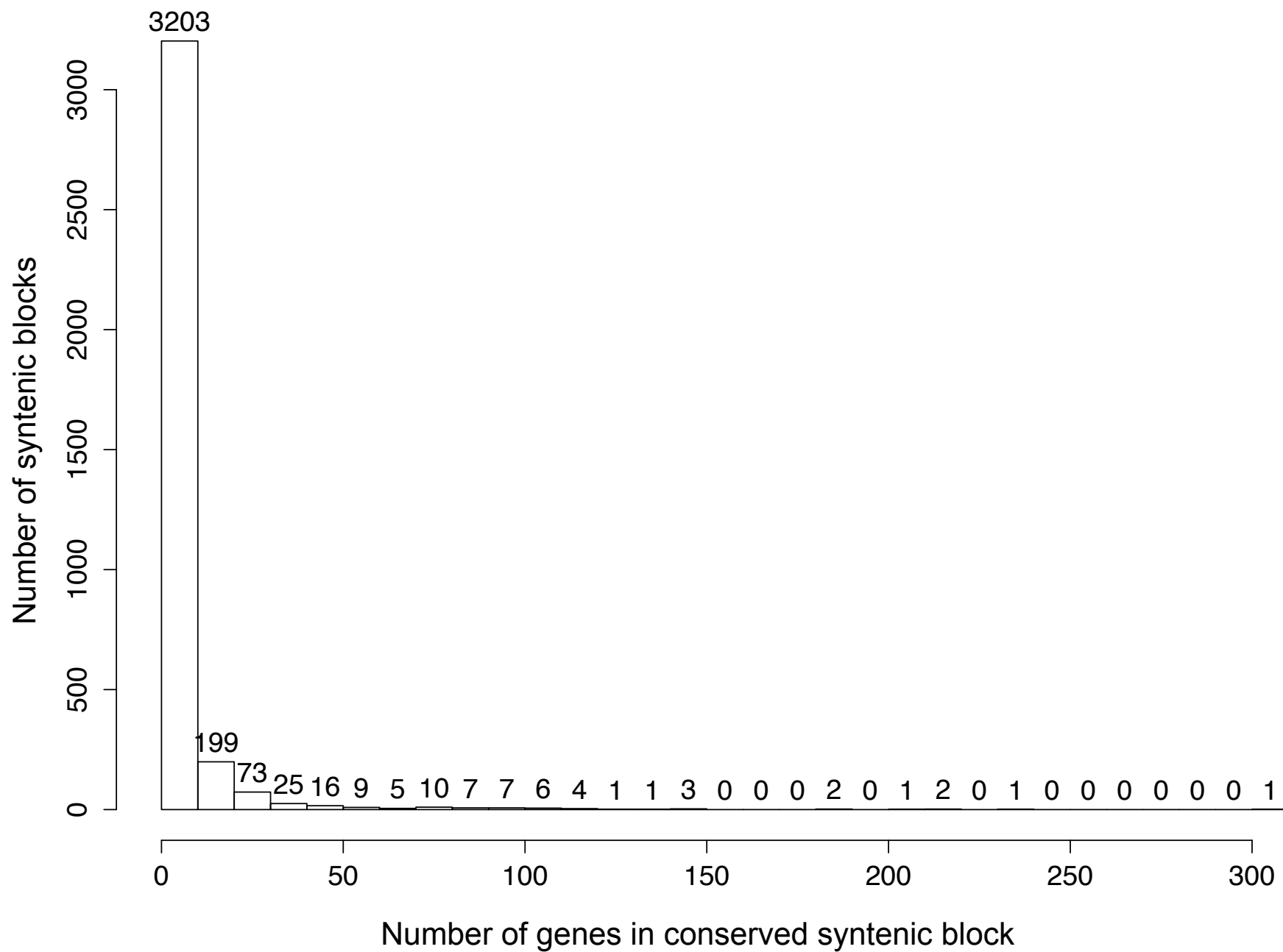

Supplement: Figure S1 — Distribution of sizes of syntenic blocks conserved between zebrafish and human. The histogram shows the number of conserved syntenic blocks containing 2–10, 11–20, 21–30 etc. genes. For this purpose, two genes are considered syntenic if they are within 100 genes from each other in both species. (PDF) [file pgen.1003734.s006.pdf]

Figure S2

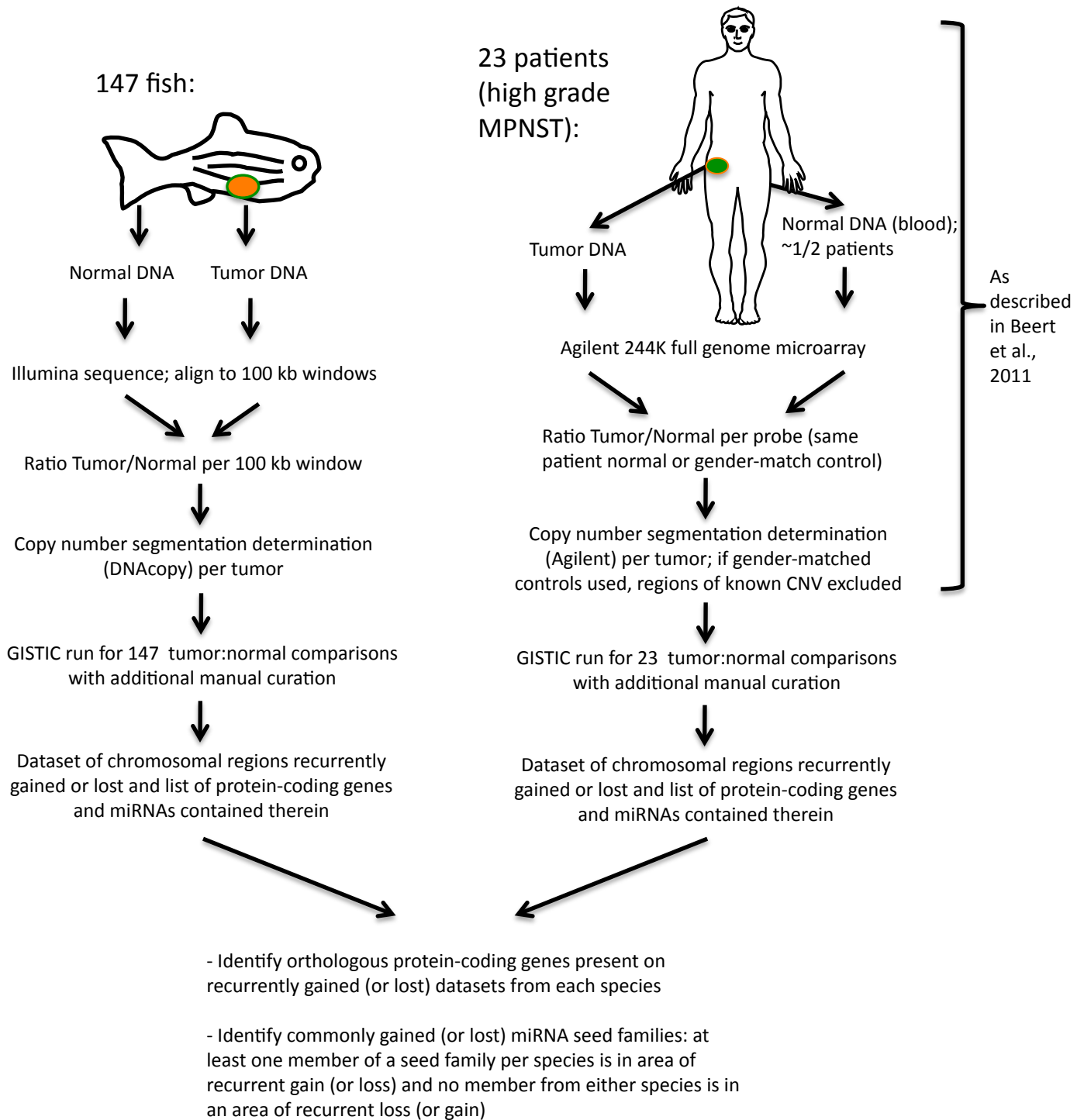

Supplement: Figure S2 — Outline of experimental approach as described in the text. (PDF) [file pgen.1003734.s007.pdf]

Figure S3

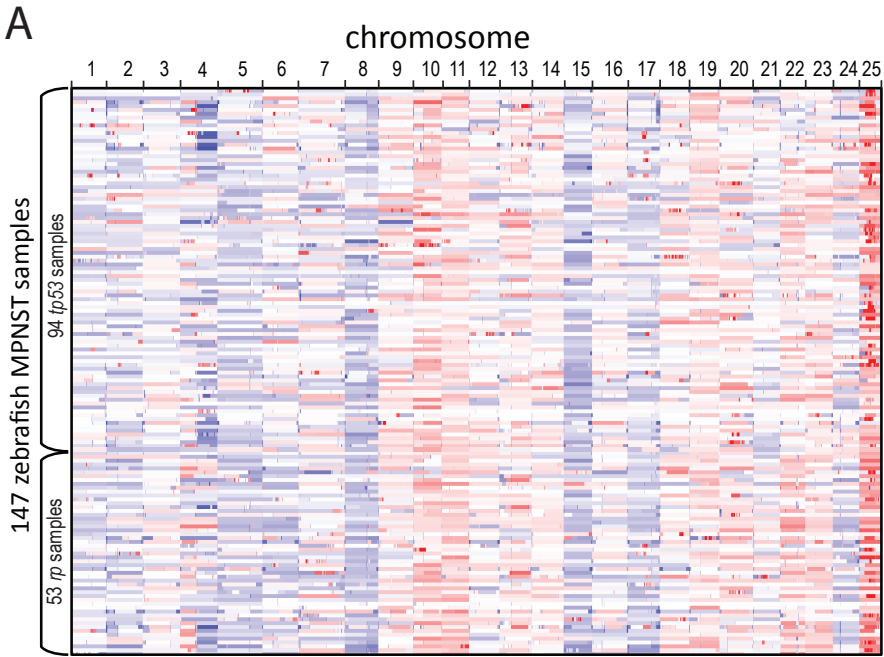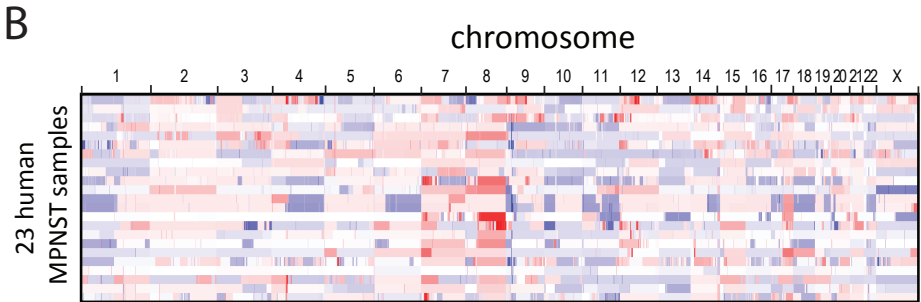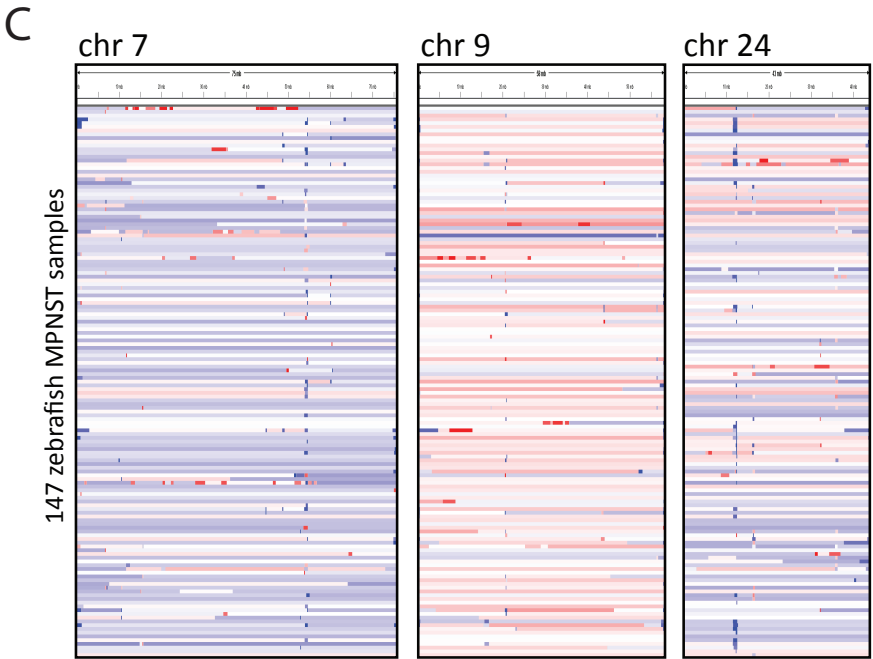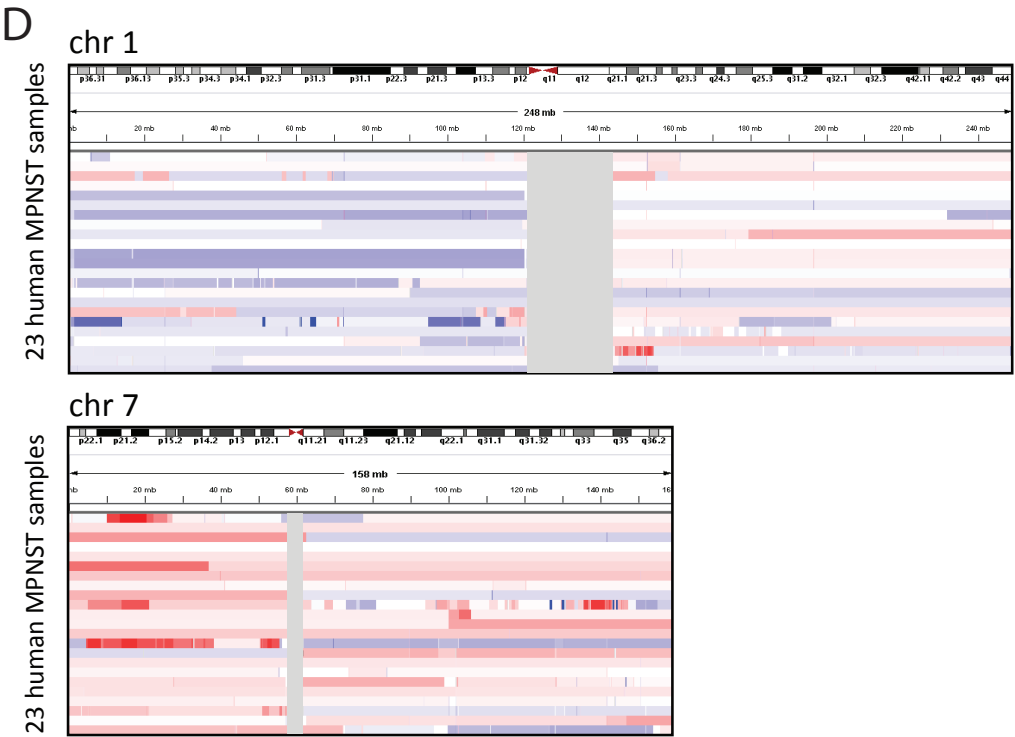

E

Zebrafish chr 1

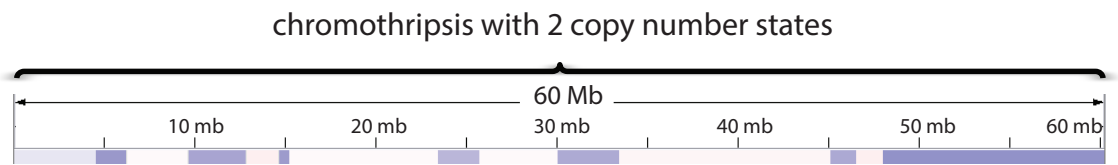

Zebrafish chr 10

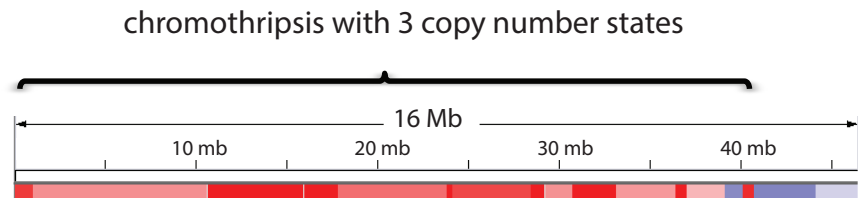

F

Human chr 5

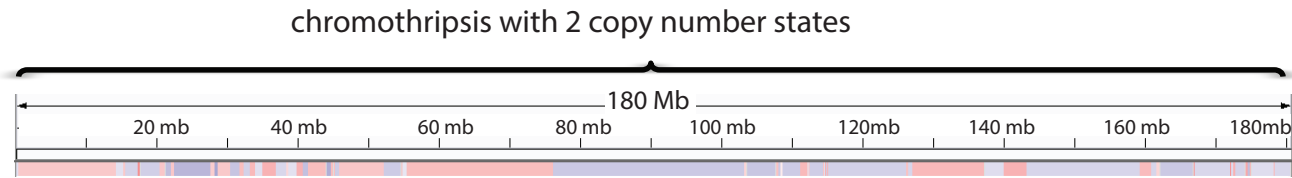

Human chr 13

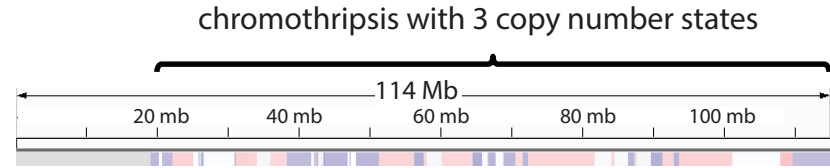

Supplement: Figure S3 — Overview and highlights of zebrafish and human MPNST CNA data. (A) Heatmap showing an overview of the CNAs for 147 zebrafish MPNST samples over chromosomes 1–25. The panel is subdivided into samples based on tp53 mutations (94 samples, top) and rp mutations (53 samples, bottom) (B) Heatmap showing an overview of the CNAs for 23 human MPNST samples over chromosomes 1–22 and X. (C) As illustrated by three examples (heatmaps for complete chromosomes 7, 9, and 24), zebrafish CNA data from MPNST samples do not suggest the existence of recurrence patterns consistent with chromosomal arms. Dashed line boxes indicate the windows within which the centromeres for these three chromosomes have been genetically mapped. (D) By contrast, and in agreement with previous studies in multiple cancer types, CNA data from human MPNSTs often reveal variability linked to chromosomal arms (chromosomes 1 and 7 shown as examples) (E, F) Repeated alternations between two or more copy number states have been described as a hallmark of chromothripsis. Such alternations, toggling either between copy number loss and neutral, between different levels of amplification, or between copy number loss and copy number gain, can be seen in the heatmaps of sample chromosomes from individual zebrafish (E) or human (F) tumors. Chromothripsis can include either a portion of a chromosome or the entire chromosome, as indicated by the brackets above each example. (PDF) [file pgen.1003734.s008.pdf]

Figure S4

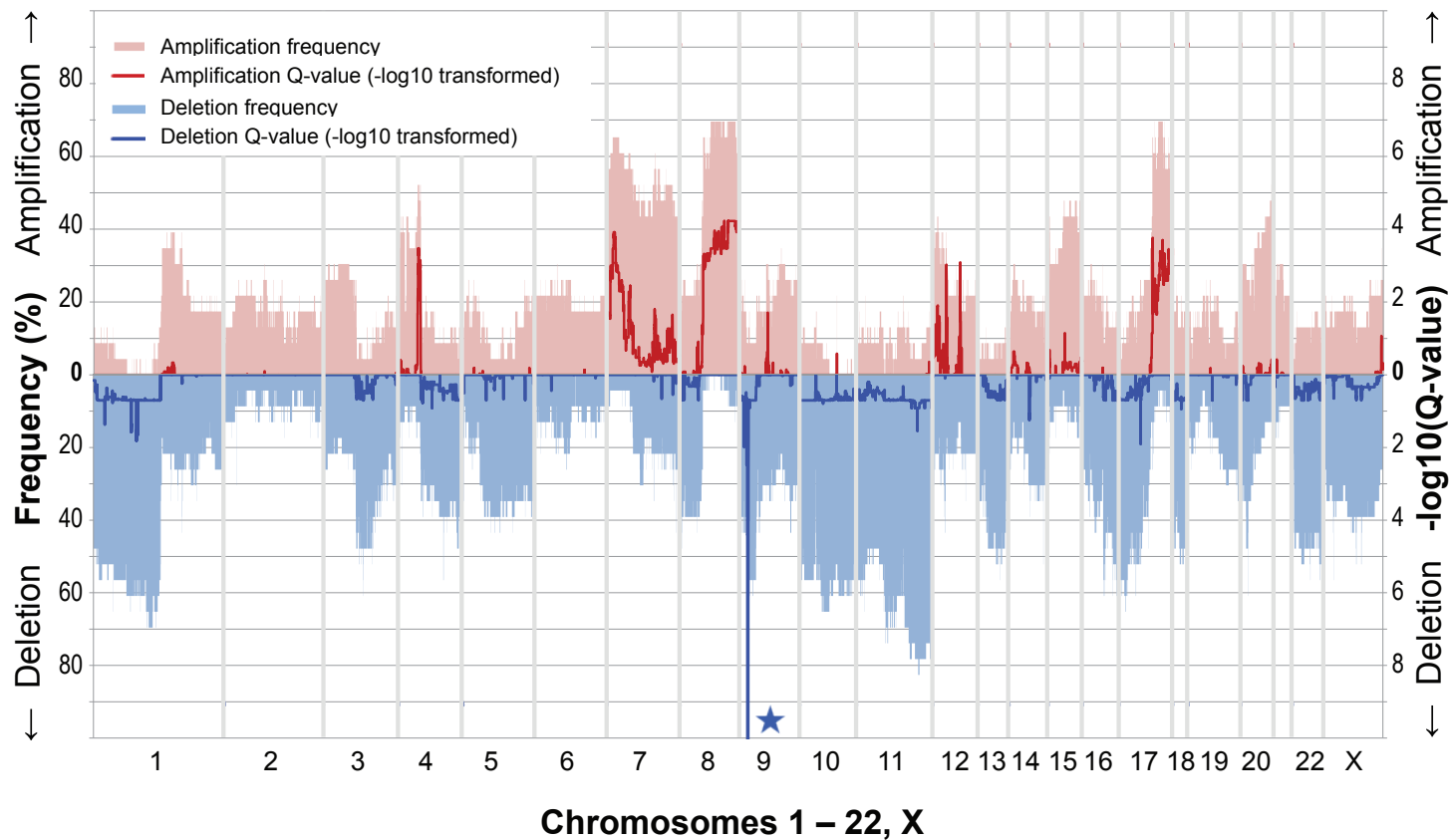

Supplement: Figure S4 — Gene-based frequency and Q-value profiles for gains (top, red) and losses (bottom, blue) over human chromosomes 1–22 and X, based on 23 MPNST samples. Gains and losses are shown in red (top) and blue (bottom). Frequencies (left y-axis, pale red/blue shading) are displayed with respect to a cutoff of 0.2 as used for the GISTIC analysis. GISTIC Q-values (right y-axis, bold red/blue lines) are displayed as −log10-transformed only above a value of 0.2 used as human-specific cutoff. The −log10-transformed Q-value for the deletion on chromosome 9 marked by a star is clipped to fit the figure and actually peaks at 20.6. (PDF) [file pgen.1003734.s009.pdf]

Figure S5

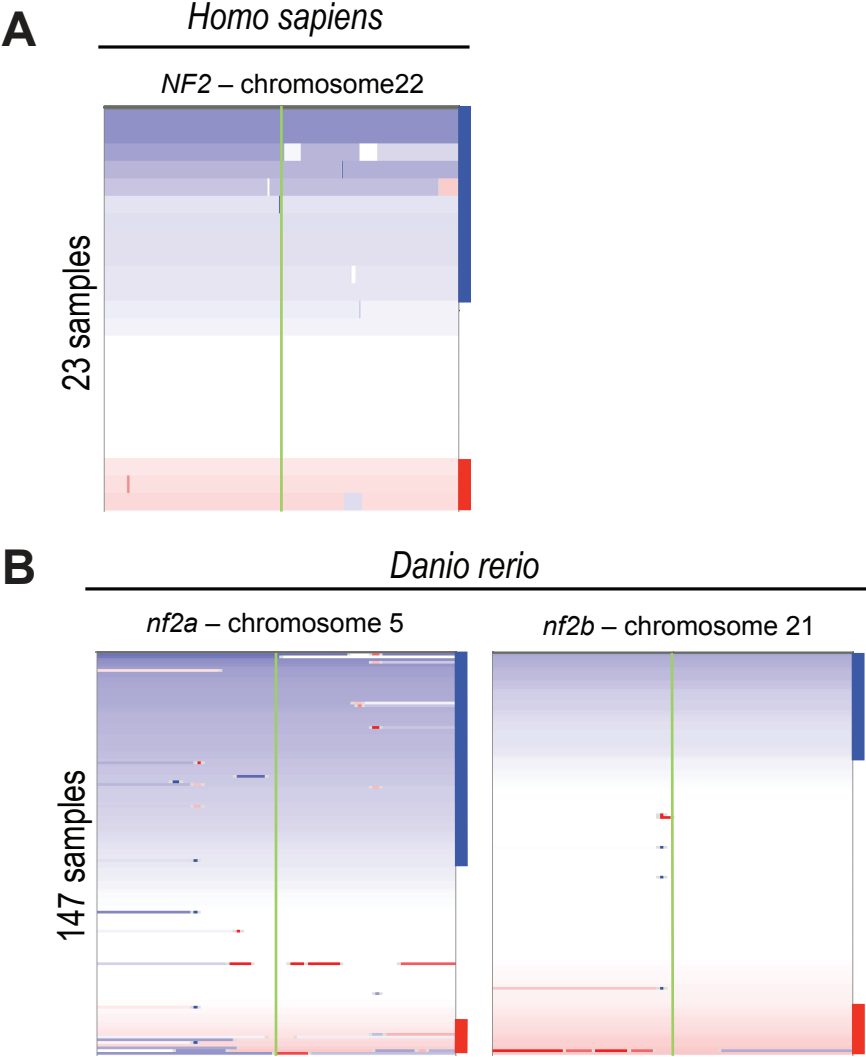

Supplement: Figure S5 — Human and zebrafish NF2 genes reside in large, not focal, CNAs. Heat maps of human (A) and zebrafish (B) CNA data showing 10 MB windows centered on NF2 loci. In each panel, samples are sorted top-to-bottom by decreasing deletion amplitude at the respective NF2 locus indicated in the center (green line). Blue and red bars at the right side of each panel indicate which samples, with respect to the NF2 locus, were actually counted as losses (blue) or gains (red) in our JISTIC analysis. (PDF) [file pgen.1003734.s010.pdf]
