# Supplementary material for: Comparative Oncogenomic Analysis of Copy Number Alterations in Human and Zebrafish Tumors Enables Cancer Driver Discovery
Source: PLoS Genet. 2013 Aug 29;9(8):e1003734. doi: 10.1371/journal.pgen.1003734 (PMC3757083; doi:10.1371/journal.pgen.1003734)
Supplement: Text S1 — Supporting Materials and Methods. (DOCX) [file pgen.1003734.s016.docx]

### *Text S1: Supporting materials and methods*

### Genomic DNA isolation, Illumina sequencing and data processing (zebrafish MPNST samples)

For every tumor, DNA was isolated from macroscopically dissected tumors and separately from normal (tail) tissue from the same fish. Based upon this paired design, CNA calls for all tumors could be determined relative to the genome of the individual fish in which it arose. Genomic DNA isolation was performed as described previously [[1](#_ENREF_1)]. Generally, sequencing and data processing was similar as described in [[1](#_ENREF_1)], with some differences in detail.

Tumor and normal DNA samples were individually processed for sequencing with the Illumina Genome Analyzer IIx system according to published methods [[2-4](#_ENREF_2)]. All of the enzymes used in the preparation were from New England Biolabs, and the oligonucleotides were synthesized by Eurofins MWG Operon. Oligonucleotide sequences (ATTGGC; GATCTG; TCAAGT; CTGATC; AAGCTA; GTAGCC; TACAAG; CGTGAT; ACATCG; GCCTAA; TGGTCA; CACTGT) were added to the 3’ end of the Illumina adaptors used for the paired-end library preparation to serve as barcodes, so that multiple samples could be sequenced in the same reaction. Sequencing was performed on an Illumina GAIIx Sequencer using Sequence Control software v2.6.26. Sequences were processed with GERALD.pl (CASAVA 1.6.0 or CASAVA 1.7.0), downloaded from the Illumina Web site (http:// www.illumina.com/software/genome_analyzer_software.ilmn), with a produced read length of 34 nucleotides. FASTQ sequence files were split into sample sets according to barcode sequences, which were then trimmed from the complete sequences using the applications fastx_barcode_splitter.pl and fastx_trimmer from the FASTX-Toolkit-0.0.13 (http://hannonlab. cshl.edu/fastx_toolkit) package. Illumina sequence qualities were converted to Sanger sequence qualities using the MAQ application fq_all2std.pl (<http://maq.sourceforge.net/qual.shtml>) [[5](#_ENREF_5)]. Sequences were then aligned to chromosomes 1-25 in the zebrafish Zv9/danRer7 assembly using bwa0.5.8c [[6](#_ENREF_6)]. Unassembled “scaffold” and “NA” fragments were not included in the alignment target. Alignments with quality scores of 10 or greater were extracted from the resulting Sequence Alignment/Map files. At this stage, individual samples were required to have at least 100,000 aligned reads, otherwise the sample and its corresponding normal or tumor counterpart were excluded from this study.

For the qualifying 294 tumor and normal samples, counts were obtained for the number of reads aligning to 13551 consecutive, adjacent 100-kb physical windows along chromosomes 1-25. This data matrix has been deposited as a supplementary file with the public GEO database record for this work (see below). The per-window counts per sample were rescaled to a total of 1 million reads (average total read count across 294 samples before rescaling = 1,226,953; average of 90 reads per 100-kb window). Log2 ratios were calculated for the matching tumor/normal sample pairs for all 100-kb windows (or set to 0, if the read count for one or both samples was 0). The log2 ratios were then submitted to the circular binary segmentation algorithm [[7](#_ENREF_7)] as implemented in the BioConductor package DNAcopy (v1.16.0), and processed with the following key parameter settings: with smoothing, undo.SD=2. 100K bin numbers were converted to genomic coordinates fixed at window midpoint, and for each sample pair, all segment mean values obtained were mean-centered before being used for further analysis. Full datasets are publicly available at the Gene Expression Omnibus database (GEO series accession 38397).

### Statistical testing for inter-subgroup differences of zebrafish MPNST samples

From processed sequencing data (log2, 100K bins), per-chromosome median values were determined for all 147 zebrafish MPNST samples for chromosomes 1 – 25 and mean-centered. For all 25 chromosomes, two t-Tests (2-tailed, homoscedastic) were performed: (i) all tumors arising in ribosomal protein gene heterozygotes (n=53) vs. all tumors arising in *p53^M214K^* homozygotes (n=94), and (ii) tumors arising in diploid *p53^M214K^* homozygotes (n=46) vs. tumors arising in triploid *p53^M214K^* homozygotes (n=48).

### Array-CGH (aCGH) data processing (human MPNST samples)

Normalized aCGH data (Agilent Feature Extraction output) for 23 human MPNST samples generated previously [[8](#_ENREF_8)], ArrayExpress database Experiment (ID: [E-MEXP-3052](http://www.ebi.ac.uk/arrayexpress/experiments/E-MEXP-3052)) was converted from log10 to log2 and submitted to the circular binary segmentation algorithm [[7](#_ENREF_7)] as implemented in the BioConductor package DNAcopy (v1.16.0), and processed with the following key parameter settings: with smoothing, undo.SD=1.

### GISTIC (JISTIC) analysis

To determine recurrent CNAs, segmented data from both zebrafish (sequencing) and human (aCGH) MPNSTs was subjected to statistical analysis using the GISTIC algorithm [[9](#_ENREF_9)] as implemented in the JISTIC software [[10](#_ENREF_10)]. JISTIC runs were performed in both standard and “focal” mode. Default parameterizations were used for both human and zebrafish, with the following exceptions: aberrationThresholds 0.2, -0.2 (zebrafish and human); genedistancemax 50000 (zebrafish) and 5000 (human), corresponding to the different effective probe density between sequencing- and array-based data. Gene collections used for the JISTIC “gene location” files were derived from Ensembl release 61 [[11](#_ENREF_11)] using both coding and non-coding genes (totaling 32,312 for zebrafish and 53,630 for human), and subsequent work (except for quality/artifact checking, see below) was exclusively performed with gene-based data.

### Converting JISTIC results into manually curated calls

The specific complexities of the zebrafish and human datasets necessitated an additional layer of manual curation, resulting in a final set of binary calls (yes or no) for recurrent large and focal copy number gains and losses, reflected in Tables S1, S3, and S4. Chief among these complexities were (i) artifacts and insufficiencies in the underlying data and (ii) our concern that the categorization into focal and large CNAs was not adequately achieved by relying on two JISTIC modes (standard and “focal”) alone. Details for how we dealt with each of these are listed below separately for the zebrafish and human data.

#### Zebrafish

Zebrafish sequencing data post-segmentation was, to a varying degree for any given tumor or normal DNA sample, sensitive to extremes in genomic GC content as described for example in [[2](#_ENREF_2)], resulting in certain artifacts. Specifically, these were short (typically, several 100K bins wide) chromosomal stretches appearing altered across many samples with near-identical boundaries, but with inconsistent polarity. Also, due to the peculiar GC content distribution in zebrafish chromosomes (generally, higher at the ends of a chromosome and gradually falling towards the center; data not shown), chromosome ends also tended to show apparent copy number alterations with inconsistent polarity. That we saw these effects in both normal and tumor samples, and did not see such phenomenon in our previous study using CGH, contributed to our assessment that these were artifacts of the sequencing technique.

These artifacts were reflected in the JISTIC results, and we compensated for them using the following approach: Artifacts were identified based on case-by-case visual comparison of the underlying segmented per-sample data, GC content, and segmented per-sample data from two other zebrafish cancer types (not shown, to be published elsewhere). Genes contained in the genomic regions deemed affected were flagged and their JISTIC Q-values (-log10 transformed) were replaced by the average of the Q-values (-log10 transformed) of the two genes adjacent on either side, or by the Q-value (-log10 transformed) of the gene on one side for artifacts at the chromosome ends.

Final recurrent CNA calls (yes or no) were then made based on the corrected JISTIC Q-values (-log10 transformed), using a cutoff of 0.6 (corresponding to a Q-value of 0.25, untransformed). Recurrent CNAs were classified as focal if they were identified either (i) in JISTIC “focal” mode or (ii) in standard mode if underlying per-sample changes were affecting a small subchromosomal range and were otherwise showing hallmarks of recurrent focal events, i.e. an apparent convergence pattern. Focal recurrent CNAs were furthermore annotated if we deemed the underlying evidence to be weak, usually due to paucity of supporting samples or supporting samples showing chromothripsis-like appearance in the region of interest (Datasets S1 and S3). Note that calls of recurrent focal CNAs may overlay those of recurrent large CNAs with the same or opposite polarity.

#### Human

Human aCGH data after segmentation showed artifacts commonly observed in array-based CNA data, namely short (50K – 500K wide) chromosomal stretches appearing altered across many samples with near-identical boundaries, but with inconsistent polarity. These artifacts were reflected in the JISTIC results, and genomic regions deemed affected based on visual inspection of the underlying per-sample data were ineligible to be called as CNAs.

Final recurrent CNA calls (yes or no) were made on the basis of JISTIC Q-values (-log10 transformed). Both the small sample count of the human dataset here and an expectation of certain CNAs based upon published reports from a number of human MPNST datasets, including the analysis of this one using a different method [[8](#_ENREF_8)], warranted a modified approach compared to the JISTIC parameters used to analyze the zebrafish dataset. We used a lower Q-value (-log10 transformed) operative cutoff of 0.2 for JISTIC standard mode, while keeping a standard cutoff of 0.6 for “focal” mode. Furthermore, on some occasions we extended calls of large recurrent CNAs through intermittent regions below cutoff; genes that were included in CNAs with sub-0.2 scores are highlighted in Table S3. Careful cross-comparison with the previous analysis of this dataset as well as with published human MPNST datasets [[12](#_ENREF_12),[13](#_ENREF_13)] convinced us that the calls were generally consistent with earlier findings for recurrent CNAs in human MPNST data.

Recurrent large and focal CNAs were differentiated in the same manner as for zebrafish (above), with the following addition: While evidence for recurrent focal CNAs was generally deemed weak (reflecting the small sample size) (Datasets S2 and S3), a few events stood out as solidly supported and were corroborated by equivalent events in another human MPNST dataset [[13](#_ENREF_13)], specifically investigated for this purpose.

### Human-zebrafish protein coding gene orthologous table construction and comparison

Only genes with Ensembl protein identifiers (release 61) mapping to assembled zebrafish chromosomes 1 – 25 and to human chromosomes 1 – 22 and X were considered. High-confidence human-zebrafish gene correspondences were established based on the approach described in [[14](#_ENREF_14)], taking advantage of conserved synteny as a guiding principle for identifying evolutionary ortholog pairs (12771 correspondences; marked “s” in Table S4, column “Category”). In order to increase coverage, we additionally considered a lower-confidence set of gene correspondences based solely on reciprocal best protein Blast hits (7728 correspondences after removing redundancy with the high-confidence set; marked “b” in Dataset S3, column “Category”). Note that in some cases, this duality results in ambiguous gene correspondences, which we left deliberately unresolved, reflecting the exploratory nature of our genome-scale approach and the fact that the extra genome duplication in the teleost lineage often results in multiple fish paralogs (ohnologs) that are orthologous to a single human gene. Finally, for simplicity, human genes from haplotype chromosome variants (6 and 17) were manually replaced with their counterparts on the respective regular chromosome (150 correspondences; marked “m” in Dataset S3, column “Category”).

After mapping the data on species-specific recurrent CNAs onto the correspondence table using Ensembl gene identifiers as a key, inter-species comparison with respect to genes affected by recurrent CNAs of the same polarity in both organisms were then performed applying straightforward Venn logic. Finally, progressing from zebrafish-human gene correspondences (Dataset S3) to human-centric gene tallies as reflected in Tables 1 and S4, a number of manual curation steps were taken: (i) obvious duplications due to partial, adjacent zebrafish gene models representing parts of the same biological gene were discounted, and (ii) correspondences where one or both partners were not of the Ensembl gene biotype “protein_coding” – i.e. immunoglobulin and T-cell receptor gene segments – were excluded.

### Human-zebrafish miRNA homologous table construction and comparison

Only genes of Ensembl gene biotype “miRNA” (release 61) from assembled zebrafish chromosomes 1 – 25 and from human chromosomes 1 -22 and X were considered. Human and zebrafish miRNA genes also present in miRBase [[15](#_ENREF_15)] (662 for human, 315 for zebrafish) were then matched using their miRBase identifiers. Matching was performed based only on the central, numeric part of the identifiers (which denotes a particular miRNA family), resulting in 89 correspondence groups containing one or more miRNAs from both human and zebrafish (Table S5B). These groups were then searched for cases where at least one member miRNA from each species was in a recurrent CNA of a certain polarity, with no member miRNAs in either species being in a recurrent CNA of the opposite polarity.

***References***

1. Zhang G, Hoersch S, Amsterdam A, Whittaker CA, Lees JA, et al. (2010) Highly aneuploid zebrafish malignant peripheral nerve sheath tumors have genetic alterations similar to human cancers. Proc Natl Acad Sci U S A 107: 16940-16945.

2. Bentley DR, Balasubramanian S, Swerdlow HP, Smith GP, Milton J, et al. (2008) Accurate whole human genome sequencing using reversible terminator chemistry. Nature 456: 53-59.

3. Quail MA, Kozarewa I, Smith F, Scally A, Stephens PJ, et al. (2008) A large genome center's improvements to the Illumina sequencing system. Nat Methods 5: 1005-1010.

4. Quail MA, Swerdlow H, Turner DJ (2009) Improved protocols for the illumina genome analyzer sequencing system. Curr Protoc Hum Genet Chapter 18: Unit 18 12.

5. Li H, Ruan J, Durbin R (2008) Mapping short DNA sequencing reads and calling variants using mapping quality scores. Genome Res 18: 1851-1858.

6. Li H, Durbin R (2009) Fast and accurate short read alignment with Burrows-Wheeler transform. Bioinformatics 25: 1754-1760.

7. Venkatraman ES, Olshen AB (2007) A faster circular binary segmentation algorithm for the analysis of array CGH data. Bioinformatics 23: 657-663.

8. Beert E, Brems H, Daniels B, De Wever I, Van Calenbergh F, et al. (2011) Atypical neurofibromas in neurofibromatosis type 1 are premalignant tumors. Genes Chromosomes Cancer 50: 1021-1032.

9. Beroukhim R, Getz G, Nghiemphu L, Barretina J, Hsueh T, et al. (2007) Assessing the significance of chromosomal aberrations in cancer: methodology and application to glioma. Proc Natl Acad Sci U S A 104: 20007-20012.

10. Sanchez-Garcia F, Akavia UD, Mozes E, Pe'er D (2010) JISTIC: identification of significant targets in cancer. BMC Bioinformatics 11: 189.

11. Flicek P, Amode MR, Barrell D, Beal K, Brent S, et al. (2011) Ensembl 2011. Nucleic Acids Res 39: D800-806.

12. Brekke HR, Ribeiro FR, Kolberg M, Agesen TH, Lind GE, et al. (2010) Genomic changes in chromosomes 10, 16, and X in malignant peripheral nerve sheath tumors identify a high-risk patient group. J Clin Oncol 28: 1573-1582.

13. Yu J, Deshmukh H, Payton JE, Dunham C, Scheithauer BW, et al. (2011) Array-based comparative genomic hybridization identifies CDK4 and FOXM1 alterations as independent predictors of survival in malignant peripheral nerve sheath tumor. Clin Cancer Res 17: 1924-1934.

14. Catchen JM, Conery JS, Postlethwait JH (2009) Automated identification of conserved synteny after whole-genome duplication. Genome Res 19: 1497-1505.

15. Kozomara A, Griffiths-Jones S (2011) miRBase: integrating microRNA annotation and deep-sequencing data. Nucleic Acids Res 39: D152-157.
